# Visuomotor Control of Human Adaptive Locomotion: Understanding the Anticipatory Nature

**DOI:** 10.3389/fpsyg.2013.00277

**Published:** 2013-05-16

**Authors:** Takahiro Higuchi

**Affiliations:** ^1^Department of Health Promotion Science, Tokyo Metropolitan UniversityTokyo, Japan

**Keywords:** walking, obstacle avoidance, adaptation, gaze behavior, older adults, optic flow

## Abstract

To maintain balance during locomotion, the central nervous system (CNS) accommodates changes in the constraints of spatial environment (e.g., existence of an obstacle or changes in the surface properties). Locomotion while modifying the basic movement patterns in response to such constraints is referred to as adaptive locomotion. The most powerful means of ensuring balance during adaptive locomotion is to visually perceive the environmental properties at a distance and modify the movement patterns in an anticipatory manner to avoid perturbation altogether. For this reason, visuomotor control of adaptive locomotion is characterized, at least in part, by its anticipatory nature. The purpose of the present article is to review the relevant studies which revealed the anticipatory nature of the visuomotor control of adaptive locomotion. The anticipatory locomotor adjustments for stationary and changeable environment, as well as the spatio-temporal patterns of gaze behavior to support the anticipatory locomotor adjustments are described. Such description will clearly show that anticipatory locomotor adjustments are initiated when an object of interest (e.g., a goal or obstacle) still exists in far space. This review also show that, as a prerequisite of anticipatory locomotor adjustments, environmental properties are accurately perceived from a distance in relation to individual’s action capabilities.

## Introduction

Locomotion, such as walking, running, cycling, or using an automobile or a wheelchair, is the behavior of moving one’s body toward a desired place. During locomotion, the critical role of the central nervous system (CNS) is not only to propel the body in the intended direction but also to maintain balance (i.e., not to fall). Balance of upright stance is ensured provided vertical projection of the center of mass (COM) falls within the base of support (BOS) (Patla, 2003). A challenging aspect of maintaining balance during locomotion is that, whereas balance during quiet stance is maintained with control of the position of COM within BOS, COM, and BOS are in motion during locomotion with BOS changing its size; during the single support phase, the size of BOS is as small as the size of one anatomical foot. Furthermore, COM during the single support phase is outside BOS; every time an individual steps with single leg, gravity-produced rolling movement of COM to the side, referred to as the lateral sway, occurs (Winter, 2004).

Another challenging aspect of maintaining balance during locomotion is that the CNS is required to accommodate changes in the constraints of spatial environment. When confronting an obstacle, for example, individuals need to control the displacement of COM to either step over the obstacle, change direction, or even stop walking. Navigating through a narrow opening requires modification of locomotor patterns if the size of the opening is too small relative to the body. Locomotion while modifying the basic movement patterns to propel in response to environmental constraints is referred to as adaptive locomotion.

To maintain balance with these challenging aspects, the CNS takes both a reactive strategy to deal with unexpected perturbation and a pre-planned strategy to avoid potential perturbation *a priori*. A pre-planned strategy is further divided into predictive and anticipatory strategies (Massion, 1992; Huxham et al., 2001; Patla, 2003; da Silva et al., 2011). A predictive strategy refers to the maintenance of inter-segmental stability within the body or between the body and surface based on the estimation of expected perturbation generated by ongoing movements. The predictive strategy is therefore used to regulate locomotion on a local level (i.e., a step-by-step basis). In contrast, an anticipatory strategy refers to the maintenance of balance on a more global level (i.e., sustained over several steps). Locomotor patterns are modified on the basis of visual information about environmental properties at a distance to avoid a future perturbation altogether.

While vision plays an important role on all of the reactive, predictive, and anticipatory strategies, the anticipatory strategy is driven exclusively by vision. This is because vision provides the spatio-temporal information regarding a remote place very precisely. Understanding the anticipatory nature of the adaptive locomotion is, therefore, particularly helpful to understand how vision is used to adaptively control our locomotion.

The purpose of the present article is to review relevant studies to reveal the anticipatory nature of the visuomotor control of adaptive locomotion. This review will yield tentative conclusions: (a) adaptive locomotion is controlled in part through anticipatory locomotor adjustments, which can be sustained over several steps; (b) while anticipatory (i.e., pre-planned) locomotor adjustments are the most powerful way to avoid perturbation, visually guided, on-line adjustments also come into play particularly in the final phase under a changeable environment; (c) a common characteristic of eye movements during adaptive locomotion is that the majority of fixations are directed either toward a desired future path or toward an object of interest; and (d) accurate visual perception of environmental properties relative to action capabilities from a remote place underlies the adaptive locomotor adjustments.

## Anticipatory Locomotor Adjustments

### Locomotor adjustments initiated at least a few steps prior to reaching an obstacle

**Figure 1:**
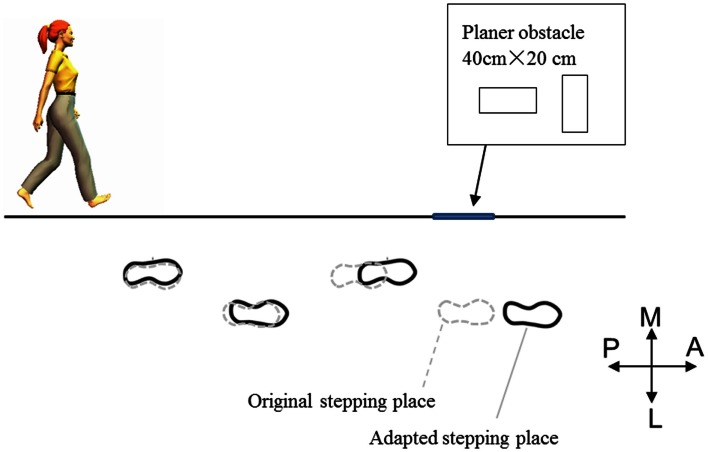
**Adapted stride length to avoid an obstacle reported in Moraes et al. (2004)**. The participants’ stride was gradually lengthened a few steps prior to reaching the planar obstacle.

When walking and encountering a specific area that would not afford stable balance, such as an icy spot on the ground, an individual would need to select an alternative foot placement to avoid stepping on that area. The dominant strategy to modulate a foot placement is to lengthen the stride to step farther from the normal landing spot (Patla et al., 1999; Moraes et al., 2004). This is understandable because it does not impede an individual’s forward progression. Importantly, the stride was gradually lengthened a few steps before they reached a spot to be avoided (Moraes et al., 2004) (Figure 1). This suggests that the adjustment of foot placement starts a few steps before reaching the area to be avoided.

When participants were asked to step over two obstacles located 1 m apart, their foot placement to take off prior to the first obstacle was closer to the obstacle than when they were stepping over a single obstacle (Krell and Patla, 2002). This is also an understandable method in order to obtain a better take-off position prior to a second obstacle and suggests that the modification of limb movement for avoiding the second obstacle was already initiated before stepping over the first one.

A similar conclusion was obtained from a study about stepping over an obstacle (Patla, 1998). The study demonstrated that even when an obstacle of height was not visible for the duration of one step prior to the participant stepping over it, the limb movements were quite similar to the condition when the obstacle was visible throughout the stepping motion (Patla, 1998). This suggests that a limb movement to step over an obstacle is already planned at least one step prior to stepping over it.

These findings clearly show the anticipatory nature of adaptive locomotor adjustments; to ensure balance at the time of avoiding an obstacle, modification of locomotor patterns are initiated at least a few steps prior to reaching it. Importantly, a decrease in movement speed has been observed prior to executing the critical locomotor adjustments such as body rotation when passing through an opening (Higuchi et al., 2006a; Cowie et al., 2010). Provided that a decrease in movement speed assists in accurately executing the critical changes in locomotor pattern (i.e., speed-accuracy trade-off), an anticipatory locomotor adjustment would be initiated much earlier than a few steps prior to reaching an object of interest.

It is likely that prior experience and knowledge about environmental constraints affect the anticipatory strategy (Huxham et al., 2001; Patla, 2003). For example, when a slip was suddenly and unexpectedly generated following foot contact on a set of steel freewheeling rollers (i.e., first-time experience of a slip in that situation), participants reactively coped with the perturbation of balance. However, after just a single experience of this unexpected slip, the participants adapted to the potential slip and modified their locomotor patterns in an anticipatory manner whenever stepping on the rollers (Marigold and Patla, 2002). This anticipatory strategy to step safely on the rollers was referred to as “a surfing strategy” by the authors, which included the attenuation of muscle response magnitude, reduced braking impulse, landing more flat-footedly, and elevating the COM. Similarly, when participants were asked to step over a fragile obstacle, they modified their limb elevation when crossing over it so that a larger spatial margin was created between the obstacle and the toe (Patla et al., 1996). This suggests that knowledge about the environmental constraints affects the anticipatory locomotor adjustments (Wagman and Malek, 2009).

### Anticipatory locomotor adjustments in a changeable environment

Advances in the understanding of adaptive locomotor control have been made by an increase in the number of investigations using changeable environmental properties (Cutting et al., 1995; Montagne et al., 2003; Fajen and Warren, 2004; Gerin-Lajoie et al., 2005; Andersen and Enriquez, 2006; Cinelli and Patla, 2008; Cinelli et al., 2008, 2009). Many of these studies showed that the anticipatory nature of adaptive step adjustments is maintained in a changeable environment. However, the strategy to adapt in a changeable environment seems to slightly different from that to adapt in a stationary environment; although an anticipatory locomotor adjustment under a changeable environment would be initiated as early as those under a stationary movement, the critical locomotor adjustments to avoid an obstacle or reach a goal are achieved in a final phase by a combination with visually guided, on-line locomotor adjustments.

Gerin-Lajoie et al. (2005) investigated how their participants circumvented an obstacle (a full-sized department store mannequin) that was initially located on a participant’s right (about 8 m apart from the participant), crossed the participant’s path at a 45° angle, and interrupted a straight walking path toward the goal (about 5 m apart from the participant). Since it is more natural to pass behind a moving obstacle (Cutting et al., 1995), the participants’ walking path was deviated to the right to pass behind the mannequin. Their initial path deviation to the right occurred about 4.5 m (approximately six steps) from the mannequin. This clearly showed that the changes in the walking path to circumvent the obstacle were planned *a priori* and initiated as soon as the participants started walking. However, the most pronounced step adjustments to deviate to the right occurred about 1.5 m from the obstacle. This suggests the importance of the final locomotor adjustments just prior to obstacle avoidance.

Cinelli et al. (2009) investigated how participants steered toward the middle of a door opening that was located 8 m from them and moved to the side as soon as they initiated walking. The main finding was that, interestingly, irrespective of whether the door opening moved to the left or right, the participants initially walked in such a way as to aim at the middle of a “doorframe,” with which the door was suspended, rather than the middle of the door opening. Cinelli et al. interpreted this finding that, when locomoting in a changeable environment, participants simplified the task by placing themselves in an area that has the greatest potential for avoiding collision (i.e., aligning themselves with the middle of the doorframe enabled them to move in either direction quickly). However, once they were in the middle of the pathway (about 2 s prior to passing through the opening), they began to aim at the middle of the door opening while looking at it. This suggests that the final locomotor adjustments were driven mainly by visually guided, on-line control, rather than by an anticipatory, pre-planned control.

When the environmental properties were continuously changing, the initial strategy to adapt was approaching while slowing down the movement (Montagne et al., 2003; Cinelli et al., 2009). When passing through moving doors that oscillated at a frequency of 1 Hz, participants were able to successfully pass through an opening by refining the regulation of their approach speed. In the final part of each walking trial, fixations were directed exclusively toward the middle of the opening. It is at this point that fine motor control is important. The coincidence of heading toward the middle of the opening and looking at that point suggests that, again, the final locomotor adjustments were likely to be driven by visually/guided, on-line control.

### Maintaining a spatial margin between an obstacle and the self

To step over an obstacle, both correct foot placement prior to “take-off” and correct limb elevation over the obstacle are required. Kinematic studies have demonstrated that for obstacles of different locations and heights, individuals can produce relatively consistent foot placement in front of the obstacle (i.e., the frontal spatial margin) and a relatively consistent toe clearance (i.e., vertical spatial margin) while stepping over it (Patla et al., 1996; Krell and Patla, 2002). This implies that maintaining a spatial margin between an obstacle and the self is one of the critical control parameters to determine how locomotor patterns were modified.

In agreement with this idea, we recently reported that when passing through an opening, the CNS is likely to determine the amplitude of body rotation to ensure that the minimal spatial margin (6–8 cm) is created at one side of the body at the time of crossing (Higuchi et al., 2012). In this study, we asked participants to walk through narrow openings of three widths relative to their body width (ratio value = 0.9, 1.0, and 1.1) while holding one of three horizontal bars (one shorter than the body width and the others 1.5 and 2.5 times the body width). The experimental manipulation of holding the long bar was helpful in addressing this issue because the longer the bar was (i.e., the wider the spatial requirements for passage were), the smaller the amplitude of body rotation sufficient to produce the same spatial margin for the respective ratio value of an opening was (see Higuchi et al., 2012 for detail). The results showed that the amplitude of rotation angles became smaller for the respective ratio value as the bar increased in length. This clearly supported the idea that producing a constant spatial margin is a control parameter for determining the amplitude of body rotations.

The magnitude of the spatial margin itself is dependent on locomotor and environmental constraints. Compared to when walking through a horizontal opening, the spatial margin for walking through a “vertical” opening (e.g., ducking to avoid a low-hanging branch) was significantly smaller (Franchak et al., 2012). Franchak et al. attributed the difference in the magnitude of spatial margin to the reflection of difference in locomotor constraints between lateral sway of the body during walking and vertical bounce; lateral sway shifts the body outside of BOS during the single support phase (Shumway-Cook and Woollacott, 2001; Fujikake et al., 2011), whereas vertical bounce only makes the body shorter (Murray et al., 1964). Likewise, when circumventing a moving obstacle, a much larger spatial margin was necessary (approximately 2 m in front and 0.5 m on each side), suggesting that environmental constraints (i.e., either a stationary or a moving environment) also affect the ideal magnitude of the spatial margin (Gerin-Lajoie et al., 2005).

## Gaze Behavior during Adaptive Locomotion

### Individuals looking at far space during adaptive locomotion

As discussed in the previous section, adaptive locomotor adjustments in response to environmental constraints, such as the existence of an obstacle are initiated when an obstacle is still far away. To assist such anticipatory adjustments, visual information about far space is necessary. Analyses of spatio-temporal patterns of gaze behavior during adaptive locomotion under a variety of environments, as well as under a variety of forms of locomotion, have shown that, except in a situation where very precise stepping on a footfall target is necessary (Hollands et al., 1995; Hollands and Marple-Horvat, 2001; Chapman and Hollands, 2006a,b; Young et al., 2012), fixations are directed toward far space.

The basic rules are that we are looking at far space and that “we are moving as we are looking” (Bernardin et al., 2012). More specifically, common characteristics of eye movements during adaptive locomotion are that the majority of fixations were directed either toward a desired future path or toward an object of interest (Land, 1999; Hayhoe and Ballard, 2005). Such a common characteristic has been observed under a variety of situations, including walking down a straight hallway to turn (Turano et al., 2001, 2002), walking through an opening (Cinelli et al., 2008, 2009; Higuchi et al., 2009a), stepping over an obstacle (Patla and Vickers, 1997), stair ascent and descent (Zietz and Hollands, 2009), stepping multiple footfall targets (Patla and Vickers, 2003; Yamada et al., 2012), steering during walking (Imai et al., 2001; Hollands et al., 2002; Lamontagne and Fung, 2009), driving a car (Land and Lee, 1994; Land and Horwood, 1995), and even walking in the dark (Grasso et al., 1998) or along mentally simulated complex trajectories (Bernardin et al., 2012).

Although common characteristics of eye movements are maintained, actual locations of fixation are different depending on whether an object of interest is on the floor. When an object of interest is on the floor, fixations tend to be directed toward the floor, particularly along a desired future path. For instance, when walking and approaching a single static obstacle located on the ground, fixations were located either at a fixed distance ahead of the individual on the floor (i.e., the direction of travel) or at the obstacle; however, fixations were never directed toward the obstacle when participants were stepping over it (Patla and Vickers, 1997). When stepping on multiple footfall targets (Patla and Vickers, 2003; Yamada et al., 2012) or going down stairs (Zietz and Hollands, 2009), individuals fixated approximately two or three targets ahead. These findings suggest that even when fixations are maintained on the floor, the rule of looking at far space is maintained. When there is no object of interest on the floor, on the other hand, fixations are rarely directed toward the floor (Turano et al., 2001, 2002; Cinelli et al., 2009; Higuchi et al., 2009a).

A somewhat exceptional case in which the rule of looking at far space is not necessarily maintained is the case of stepping very precisely on a footfall target (Hollands et al., 1995; Hollands and Marple-Horvat, 2001; Chapman and Hollands, 2006a,b; Young et al., 2012). In such cases, individuals look at the footfall target on which they intend to step until they step on the intended target. This suggests that on-line visual information is necessary to step very precisely on a footfall target. Importantly, however, fixation patterns in this case are still the same as those in other cases in that individuals are likely to rely on the maintained fixations directed toward goal-oriented locations; that is, individuals are aiming at where they are looking (Bernardin et al., 2012).

### The use of optic flow

Maintaining fixation at a distant point on (or very close to) a desired future path helps individuals to align themselves with the goal (Hollands et al., 2002; Wilkie and Wann, 2003; Marple-Horvat et al., 2005) because such fixations simplify control of the heading direction through reliance on optic flow (Warren et al., 2001; Andersen and Enriquez, 2006). Optic flow is the retinal motion pattern generated by body movement (Gibson, 1958; Warren et al., 2001). When an individual fixates on a point, its location on the retina remains stationary while motion radiates from the point with the maximum velocity to the side. Gibson (1958) called this stationary point the focus of expansion (FoE) of the optic flow field. When traveling in a straight line, the current direction of motion is specified by the FoE, so in principle the heading direction can be accurately controlled by ensuring that the FoE always lies in the desired path (Wilkie and Wann, 2003).

**Figure 2:**
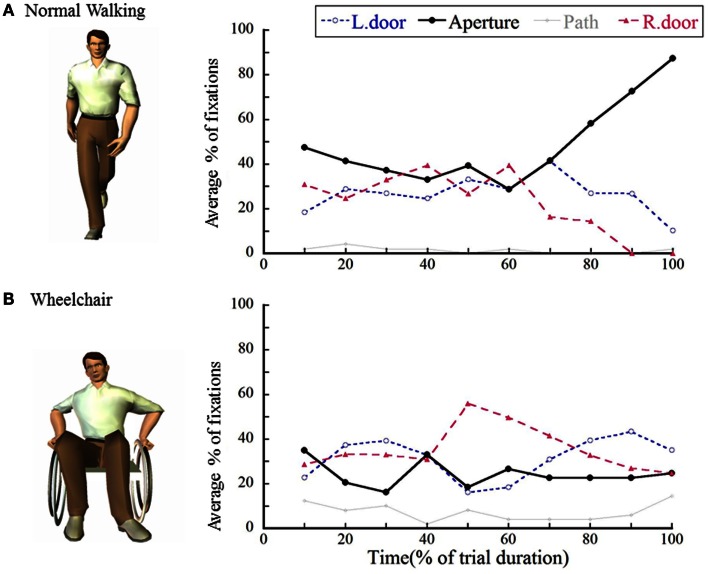
**Average percentages of fixations directed toward each of the four possible locations at each interval for (A) normal walking and (B) wheelchair conditions**. The value on the *x* axis shows the normalized time of trial (0% corresponds to the initiation of the trial, and 100% corresponds to the time of crossing). This figure is reproduced with permission from Higuchi et al. (2009a).

Figure 2A shows average percentages of fixations directed toward each of the four locations [left door, aperture, floor (path), or right door] while approaching and crossing a narrow opening (Higuchi et al., 2009a). As already explained in the previous section (Cinelli et al., 2009), fixations were directed exclusively toward the middle of the opening in the final part of each walking trial (for the last 10% of the normalized walking time). This finding is very important and suggests that even for stationary obstacles, visually guided, on-line control with the use of optic flow will come into play at the final phase of avoiding a collision.

### Alternative explanations for the function of gaze behavior

Alternative explanations for the functions of observed fixation patterns are also possible. First, by directing their fixations toward a desired future path or an object of interest, individuals could have been using their peripheral visual field to search for potential collision or perturbation. When passing through an opening, for example, maintaining a fixation toward the opening may have served as “visual pivot” (Ripoll et al., 1995; Williams et al., 1999) so that both sides of the doors with which collision could occur were captured in their peripheral vision, leading to the safest navigation through an opening (Cinelli et al., 2009).

Second, considering that common characteristics of eye movements during adaptive locomotion are maintained even when walking in the dark (Grasso et al., 1998), stepping on an invisible footfall target (Hollands and Marple-Horvat, 1996), or along mentally simulated complex trajectories (Bernardin et al., 2012), the brain uses the corollary motor command to the eye as a feed-forward signal to guide the expected direction. The efferent information about motor commands and proprioception given by eye muscles when modifying their direction provides an important non-visual source of information. The fixation location during locomotion may therefore be necessarily aligned with a desired future path. Studies regarding eye-hand coordination during manual aiming tasks support this explanation (Abrams et al., 1990; Wilmut et al., 2006).

Notably, at least for passing through a narrow opening, spatio-temporal patterns of fixation are dramatically different when the form of locomotion is quite novel for participants (Higuchi et al., 2009a). Figure 2B shows that when participants were naïve to wheelchair use and they tried to pass through an opening while sitting in a wheelchair, fixations were directed more frequently toward the door edges throughout their locomotion. At the same time, the duration of each fixation became significantly shorter. By foveating the door edges, the participants were better able to attend to the doors’ positions, while short fixation durations allowed the participants to process each door’s location more frequently. The differences in spatio-temporal patterns of fixation while walking or using a wheelchair seem to be similar to those between elite and non-elite athletes (Kato and Fukuda, 2002; Martell and Vickers, 2004; Nagano et al., 2004; Panchuk and Vickers, 2011), in that non-elite participants showed shorter fixation and more frequent saccades at critical moments.

It appears that without a great deal of locomotor experience with a wheelchair, participants were unable to adapt to locomotor constraints imposed during wheelchair use and/or to effectively use optic flow to guide wheelchair locomotion. Attributing the specific patterns of fixation under the wheelchair condition to unfamiliarity with wheelchair use is indirectly supported by the findings demonstrating that a great deal of practice is necessary to effectively use optical variables in motor control (Michaels and de Vries, 1998; Jacobs et al., 2001; Fajen and Devaney, 2006). This is referred to as perceptual attunement. The existence of perceptual attunement has been demonstrated with perceptual-motor tasks, such as judging optic angles or the expansion rate of an approaching ball (Smith et al., 2001). It seems likely that similar learning process is necessary to effectively use optical variables during adaptive locomotion.

### Maladaptive gaze behavior in older adults who are at high risk of falling

**Figure 3:**
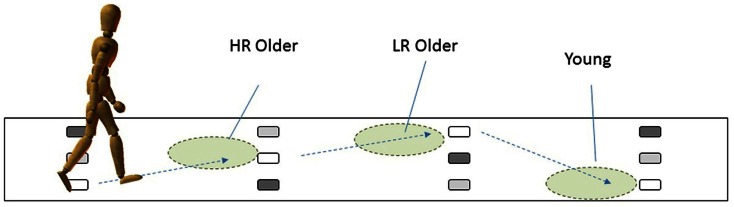
**Group differences in how far ahead the participants fixated while performing the MTS task**. Compared to the younger participants, who generally fixated three steps ahead, older participants showed the tendency to fixate on/around an imminent footfall target. Such a tendency was stronger for those who were categorized as high risk (HR) older participants than for those who were categorized as low risk (LR) older participants. This figure was produced on the basis of the report in Yamada et al. (2012), and reproduced with permission from Higuchi et al. (2013).

We recently developed a new assessment for the fall risk of older individuals, the multi-target stepping (MTS) test, to measure stepping accuracy in a simplified manner (Yamada et al., 2011). In the MTS test, participants were asked to walk while stepping on multiple footfall targets and avoiding non-targets. In one of the studies to validate the MTS test (Yamada et al., 2012), we compared gaze behaviors while performing the MTS test among the three groups: the older individuals who are at high risk (HR) of falling (HR older), those who are at low risk (LR) of falling (LR older), and young individuals. The results showed that whereas the younger participants fixated approximately three targets ahead, the older participants directed their fixation closer toward the imminent footfall target. Such a tendency was significantly higher for the HR older participants than the LR older participants (Figure 3). Furthermore, the closer toward the imminent footfall target their fixations were, the more frequent were the errors of failure in stepping on the target and of avoiding non-targets.

From these findings, it is suggested that HR older individuals may have tried to precisely step on multiple footfall targets by heavily relying on on-line, visual information about an imminent footfall target; as a result, they were unable to modify their locomotor pattern in an anticipatory manner, resulting in more frequent stepping errors. HR older individuals generally exhibit increased gait variability (Verghese et al., 2009; Brach et al., 2010), a decline in visuomotor control of foot movement (Chapman and Hollands, 2006b, 2007). For these reasons, relying deeply on visually guided, on-line control of foot movement until they step on an imminent footfall target may have been unavoidable.

## Perception of Environment in Relation to Action Capabilities

### Optical variables as a fundamental basis for guidance of locomotion

The fundamental basis for the guidance of locomotion is the patterned distribution of light available at a moving point of observation. As discussed, this patterned distribution experienced at the retina has been commonly referred to as optic flow (Gibson, 1958, 1979) or optical variables (Warren, 1998; Fajen, 2001). A considerable amount of research has been conducted to identify both the properties of optical flow that might support the guidance of locomotion and strategies that humans and animals use to exploit these properties of optic flow to achieve locomotor tasks.

For example, the time until contact with an object toward which one is moving at constant velocity happens to equal the inverse of the rate of dilation of the closed optical counter of the object (Lee, 1976). It is possible to tell when an object will be contacted by determining the rate at which its image expands (Rosenbaum, 2010). One can use this well-known tau-dot strategy for regulating deceleration during braking (Lee and Thomson, 1982; Lamontagne et al., 2007). Similarly, one behaves similarly to bees in that both steer down the middle of a passageway by equating the speed of optic flow (Duchon and Warren, 2002).

One can also align the direction of locomotion with the goal by turning by an amount that corresponds to the visual angle between the FoE and the goal (Harris and Carre, 2001; Warren et al., 2001; Lamontagne et al., 2010; Li and Cheng, 2011). Steering toward a goal requires that observers null the heading angle before reaching the target. That is, steering may be thought of as coordinating the closure of the two gaps: the heading and between the observer and the target. This strategy has been referred to as the tau-equalization theory (Fajen, 2001). Central vision is likely to be important for using optic flow to guide walking (Turano et al., 2005).

### The necessity of body-scaled (or action-scaled) information for adaptive locomotion

As explained, individuals generally rotate their body when an opening is narrower than 1.1–1.3 times their shoulder width (Warren and Whang, 1987; Higuchi et al., 2006a, 2012; Franchak et al., 2012). This rotation is initiated generally two steps prior to entering the opening (Higuchi et al. in an unpublished data) and its amplitude is determined so that it produces a minimum spatial margin under safe situations (Higuchi et al., 2012). The prerequisite of such behavior is that individuals can perceive “the width of an opening relative to the body width,” or more generally, “the environmental properties relative to one’s action capabilities” very accurately when the opening is still far from them. The perception of the fit between a person’s action capabilities and relevant environmental properties has generally been referred to as perception of affordances (Gibson, 1979).

The information of the environmental properties relative to one’s action capabilities is often referred to as body-scaled (or action-scaled) information (or more simply, the critical ratio value). Not only scaling body rotation angles but also other locomotor modifications when navigating through openings, such as changes in speed (Higuchi et al., 2006a; Cinelli et al., 2008; Cowie et al., 2010; Fajen and Matthis, 2011) or the magnitude of deviation of the body midline from the center of the apertures (Higuchi et al., 2006a; Nicholls et al., 2010; Fujikake et al., 2011), were also well proportioned to this critical ratio value. These findings lead researchers to a general understanding that the perception of the ratio value be important to control gait and posture for navigating through apertures (Warren and Whang, 1987; Wagman and Taylor, 2005; Higuchi et al., 2006a; Fajen and Matthis, 2011). The validity of this understanding has been strengthened by another line of studies demonstrating that body-scaled (or action-scaled) information is also used to estimate a maximum reach (or jump-reach) height (Ramenzoni et al., 2008, 2010; Wagman and Morgan, 2010), a maximum height of surface that can be sat on (Mark, 1987; Mark et al., 1990) a maximum inclined surface that afford standing (Regia-Corte and Wagman, 2008), or stepping over a gap (Burton, 1992; Jiang and Mark, 1994; Snapp-Childs and Bingham, 2009).

### Recalibration in response to altered action capabilities

Action capabilities are not always constant in daily locomotor activities; they are altered when walking while holding a shopping bag or a large box. Since a wider space than usual is required for locomotion under these situations, the dimensions of an external object needs to be accurately represented by the CNS as if it were a biological extension of the body. In other words, body-scaled (or action-scaled) information needs to be recalibrated in response to the extension (Higuchi et al., 2006b).

Previous studies showed an individual’s superior ability to adapt to artificial extensions of body dimensions while walking (Mark, 1987; Mark et al., 1990; Hirose, 2002; Higuchi et al., 2006a) or while judging whether an aperture is passable with the extensions (Wagman and Taylor, 2005; Wagman and Malek, 2007). Higuchi et al. (2006a) demonstrated that when rotation of the shoulders was free at the time of door crossing, participants were very successful in crossing a doorway while holding a 63-cm horizontal bar; virtually the same locomotor patterns as those during normal walking were observed.

However, an individual’s superior ability to quickly adapt to artificial extensions seems to occur only for well-learned behavior. In one of our studies (Higuchi et al., 2004), we demonstrated that young, non-handicapped participants who had never used a wheelchair underestimated the space required for a wheelchair, risking a potential collision. They determined that apertures would be passable when apertures were greater than 0.94 times the width of the wheelchair. Their underestimation was not completely eliminated even after 8 days of practicing moving through openings of various widths with a wheelchair. These findings suggest that it would take a long time to adapt to altered action capabilities while using a wheelchair. Since the biomechanical features of locomotion dramatically change from walking to wheelchair use (e.g., upper-limb propulsion, restricted mobility, and dramatic changes in the position of the COM and BOS), extensive practice may be required to accurately determine whether safe passage is possible. In fact, the estimation of the space required for wheelchair use was accurate in experienced users with tetraplegia (Higuchi et al., 2009b) and healthy participants trained for 6 months (Flascher and Shaw, 1995).

Moreover, an individual’s superior ability to quickly adapt to artificial extensions under a specific context, which is obtained through extensive practice, is not necessarily transferred under a novel context. Players of American football, who have had extensive practice in running through narrow spaces while wearing large shoulder pads, exhibited greater efficiency in running through narrow apertures than control athletes (Higuchi et al., 2011). Specifically, they exhibited smaller magnitudes and later onset times of body rotations than the control athletes. Importantly, however, such differences occurred only when they were running through openings and not while they were walking through openings. The results highlight that their excellent ability to quickly adapt to artificial extensions while wearing the shoulder pads is context specific (i.e., speed dependent).

Age-related changes in adaptability to altered action capabilities have also been reported (Hackney and Cinelli, 2013). Hackney and Cinelli initially demonstrated an age-related difference in behavior when walking through apertures; older adults were likely to adopt a more cautious strategy when passing through (i.e., creating a wider spatial margin). They then showed that affordance perception for aperture passability was less accurate for older participants only when the perception was made while they were in motion. The authors concluded with the finding that, for older adults, affordance perception is affected by self-motion, which could carry over to their locomotion.

## Concluding Remarks

Understanding the anticipatory nature of adaptive locomotion is helpful in understanding how vision is used to adaptively control our locomotion. This is because vision exclusively provides the information regarding a remote place very precisely. This paper reviewed a number of studies that have shown the anticipatory nature of adaptive locomotion. To ensure balance at the time of avoiding an obstacle, modifications in locomotor patterns are initiated at least a few steps prior to reaching the obstacle. It seems likely that maintaining a consistent but minimum spatial margin between an obstacle and the self is one of the dominant control parameters in determining how locomotor patterns are modified. Particularly, to avoid moving obstacles, not only executing anticipatory locomotor adjustments when obstacles are still far away but also making visually guided, on-line locomotor adjustments in the final phase is necessary. Eye movement during adaptive locomotion is well suited to assisting anticipatory locomotor adjustments. The basic rules are that we are looking at far space and that “we are moving as we are looking” (Bernardin et al., 2012). The CNS is smart enough to perceive environmental properties relative to action capabilities. The CNS is also flexible enough to recalibrate the perception in response to altered action capabilities, although the recalibration seems to occur very quickly only for well-learned behavior. Finally, inability to rely on anticipatory strategy to control adaptive locomotion with age can result in increased fall risk. Future studies need to examine whether balance problems during locomotion in some types of patients, such as stroke patients or patients with Parkinson’s disease, can also be explained in part with their inability to rely on anticipatory strategy to control adaptive locomotion.

## Conflict of Interest Statement

The authors declare that the research was conducted in the absence of any commercial or financial relationships that could be construed as a potential conflict of interest.
